# KLF5 promotes cervical cancer proliferation, migration and invasion in a manner partly dependent on TNFRSF11a expression

**DOI:** 10.1038/s41598-017-15979-1

**Published:** 2017-11-16

**Authors:** Dong Ma, Ling-Ya Chang, Shan Zhao, Jun-Jian Zhao, Yan-Jie Xiong, Fu-Yuan Cao, Lu Yuan, Qi Zhang, Xin-Yue Wang, Mei-Li Geng, Huan-Yu Zheng, Ou Li

**Affiliations:** 1 School of Public Health, North China University of science and technology, Jianshe Road 57, Tangshan, 063000 Hebei China; 2 Department of Cancer Second Division, The Second Hospital of Hebei Medical University, 215 Heping West Road, Shijiazhuang, 050000 Hebei China; 3 North China University of Science and Technology Affiliated Hospital, Jianshe Road 57, Tangshan, 063000 Hebei China; 4 Department of Obstetrics and Gynecology, Workers’ Hospital of Tangshan, Wenhua road 27, Tangshan, 063000 Hebei China

## Abstract

Although the transcription factor Krüppel-like factor 5 (KLF5) plays important roles in both inflammation and cancer, the mechanism by which this factor promotes cervical carcinogenesis remains unclear. In this study, we demonstrated a potential role for tumour necrosis factor receptor superfamily member 11a (TNFRSF11a), the corresponding gene of which is a direct binding target of KLF5, in tumour cell proliferation and invasiveness. Coexpression of KLF5 and TNFRSF11a correlated significantly with tumorigenesis in cervical tissues (*P* < 0.05) and manipulation of KLF5 expression positively affected TNFRSF11a mRNA and protein expression. Functionally, KLF5 promoted cancer cell proliferation, migration and invasiveness in a manner dependent partly on TNFRSF11a expression. Moreover, *in vivo* functional TNFRSF11a-knockdown mouse studies revealed suppression of tumorigenicity and liver metastatic potential. Notably, tumour necrosis factor (TNF)-α induced KLF5 expression by activating the p38 signalling pathway and high KLF5 and TNFRSF11a expression increased the risk of death in patients with cervical squamous cell carcinoma. Our results demonstrate that KLF5 and TNFRSF11a promote cervical cancer cell proliferation, migration and invasiveness.

## Introduction

Cervical cancer (CC) is a major cause of cancer-related deaths in women worldwide, accounting 250,000 deaths each year^1^. However, effective therapies for this deadly disease are limited because the elaborate molecular mechanism underlying CC progression remains largely unknown^2,3^. Several reports have suggested links between the aggressive nature of human cervical carcinoma and a number of molecular abnormalities, including the inactivation of various tumour suppressor genes and activation of various oncogenes^4,5^. This lack of sufficient genetic and epigenetic data regarding the pathogenesis of CC and the paucity of effective targets has hindered the development of novel targeted therapies^6–8^.

Krüppel-like factor 5 (KLF5) is a DNA-binding transcriptional regulator^9^ that contributes to the regulation of various cellular processes, including cell proliferation, differentiation, angiogenesis and migration^10–13^, by regulating several important target genes, such as platelet-derived growth factor (PDGF)-α^14^, cyclinD1^15,16^, survivin^17^, p21^18^ and p27^19^. KLF5 has been reported to play opposing roles in tumorigenesis; some studies^20^ have described a tumour suppressive role, whereas others cite a tumorigenic role^21,22^. This binary nature is unusual in the setting of carcinogenesis, and the mechanisms that control the functional switching of KLF5 seem to be context-dependent^15,23^. In keratinocytes, KLF5 promotes cell migration by inducing the transcription of integrin-linked kinase^24^. However, the mechanism by which KLF5 exerts its effects has not been elucidated in the context of CC cell migration and invasion.

Tumour necrosis factor receptor superfamily member 11a (TNFRSF11a) is a type I homotrimeric transmembrane protein that shares the highest level of homology with CD40^25^. TNFRSF11a is expressed widely^26^ in the heart, lung, brain, skeletal muscle, kidney, liver and skin^25,27^, as well as some cancers^28^, including breast and prostate cancers^29,30^ which possess a high bone metastasis potential. In a previous study of mice, TNFRSF11a-mediated intracellular signalling was found to be essential for mammary gland development by regulating the expansion of the stem and progenitor cell compartments. Conversely, TNFRSF11a overexpression in mice promoted abnormal proliferation and impaired differentiation, thus increasing the incidence of tumorigenesis^31^. A potential role for TNFRSF11a in tumour cell proliferation is being investigated; if proven, this molecule could be a future target of anti-tumour therapies^29^. However, the regulatory functions and mechanisms of TNFRSF11a in CC are largely unknown.

We hypothesised that KLF5 might promote tumorigenesis in CC tissues in part by directly regulating *TNFRSF11a* transcription. In this study, we demonstrated that both TNFRSF11a and KLF5 were strongly expressed in HeLa and SiHa cells and human cervical squamous cell carcinoma (CSCC) tissues. KLF5 directly bound to the *TNFRSF11a* promoter to induce transcription and in turn promoted CC cell proliferation and migration *in vitro*. Notably, the p38 signalling pathway mediated the effects of KLF5 and TNFRSF11a on CC cell proliferation and migration. These findings provide evidence to support an expression-based and functional link between KLF5 and TNFRSF11a and shed light on the regulation of TNFRSF11a and its functions in CC.

## Results

### KLF5 and TNFRSF11a are coexpressed in cervical tissues and cell lines

**Figure 1:**
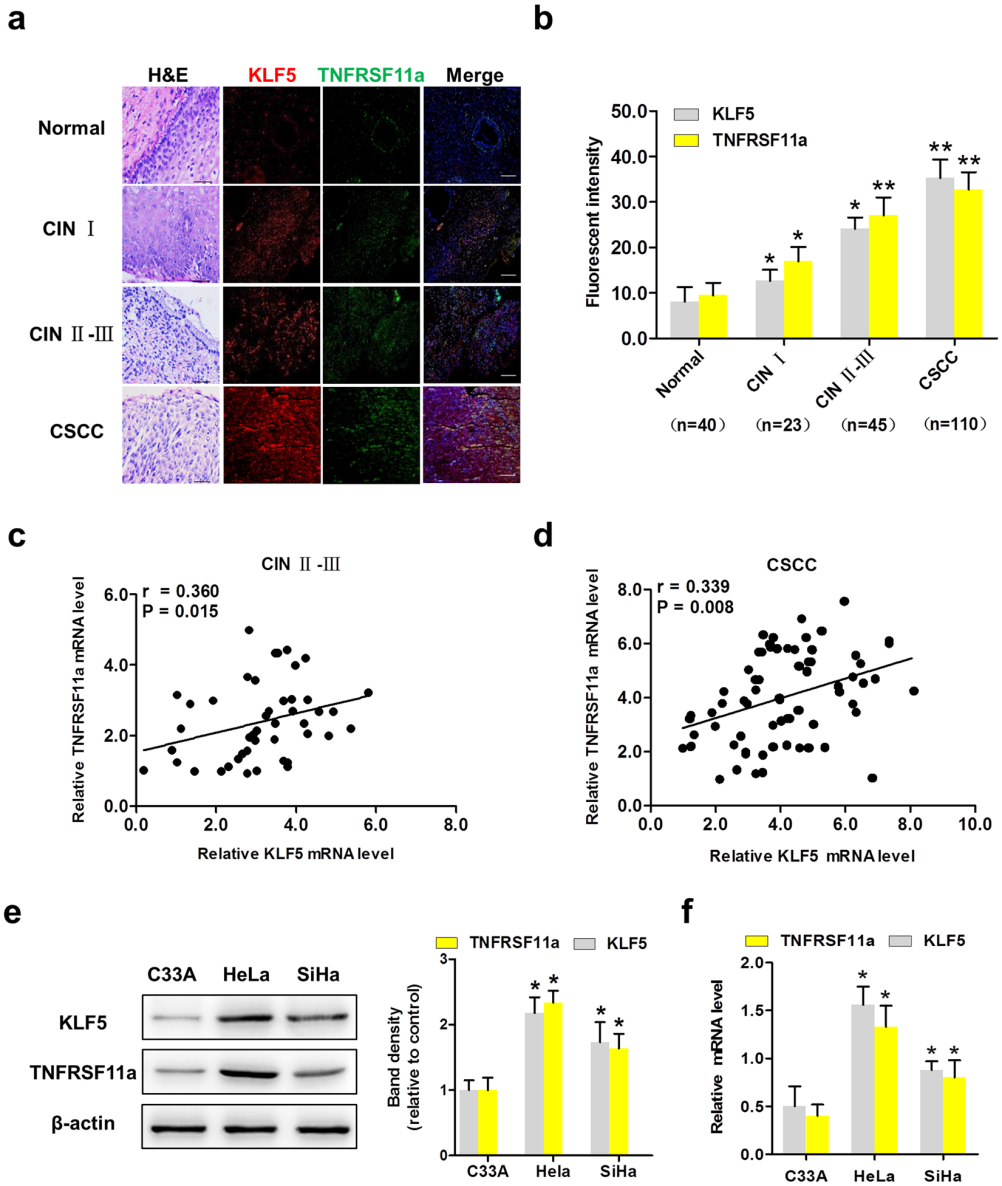
Krüppel-like factor 5 (KLF5) and tumour necrosis factor receptor superfamily member 11a (TNFRSF11a) are coexpressed in cervical cancer tissues and cell lines. (**a**) Haematoxylin–eosin and immunofluorescence staining of KLF5 (red) and TNFRSF11a (green) in cervical tissues (40 normal cervical tissues, 68 cervical intraepithelial neoplasia (CIN) tissues (CIN I: 23 cases; CIN II-III: 45 cases) and 110 CSCC tissues) (×200). Scale bars = 200 μm. (**b**) Statistical analysis of fluorescence intensity. *P < 0.05, **P < 0.01 vs. Normal group. (**c**) KLF5 mRNA expression correlated significantly with TNFRSF11a mRNA expression in 45 patients with CIN II-III. (**d**) KLF5 mRNA expression correlated significantly with TNFRSF11a mRNA expression in 110 patients with cervical squamous cell carcinoma (CSCC). Expression of KLF5 and TNFRSF11a at the protein level (**e**) and mRNA level (**f**) in C33A, HeLa and SiHa cells. *P < 0.05 vs. C33A cells.

To determine whether both molecules were responsible for cervical tumorigenesis, we examined their expression in cervical tissues using immunofluorescence (IF) and haematoxylin–eosin staining. As shown in Fig. 1a, dual staining revealed that the fluorescence intensities of KLF5 (red) and TNFRSF11a (green) were distributed differently among various cervical tissues, including normal tissues (The fluorescent intensity: KLF5: 8.08 ± 3.17 and TNFRSF11a: 9.46 ± 2.76, n = 40), cervical intraepithelial neoplasia (CIN) I tissues (KLF5: 12.65 ± 2.46 and TNFRSF11a: 16.96 ± 3.14, n = 23), CIN II-III tissues (KLF5: 24.09 ± 2.53 and TNFRSF11a: 27.05 ± 3.95, n = 45) and CSCC tissues (KLF5: 35.33 ± 3.97 and TNFRSF11a: 32.72 ± 3.85, n = 110) (all P < 0.05, Fig. 1b). Finally, we analysed the levels of KLF5 and TNFRSF11a mRNA in 45 CIN II-III samples (Fig. 1c) and 110 CSCC samples (Fig. 1d) and observed that a significant correlation of TNFRSF11A with KLF5 expression in the latter. These results suggest that KLF5 and TNFRSF11a are coexpressed in CIN II-III and CSCC tissues and correlate with the cervical carcinogenesis. To further understand of KLF5 and TNFRSF11a is associated with cervical cancer progression. A western blotting analysis revealed significantly increased KLF5 and TNFRSF11a protein expression in SiHa and HeLa cells, compared with C33A cells (Fig. 1e). A quantitative real-time polymerase chain reaction (qRT-PCR) assay revealed higher mRNA levels in SiHa and HeLa cells, compared to C33A cells (Fig. 1f).

### KLF5 mediates the tumour necrosis factor (TNF)-α-induced proliferation, migration and invasion of CC cells

**Figure 2:**
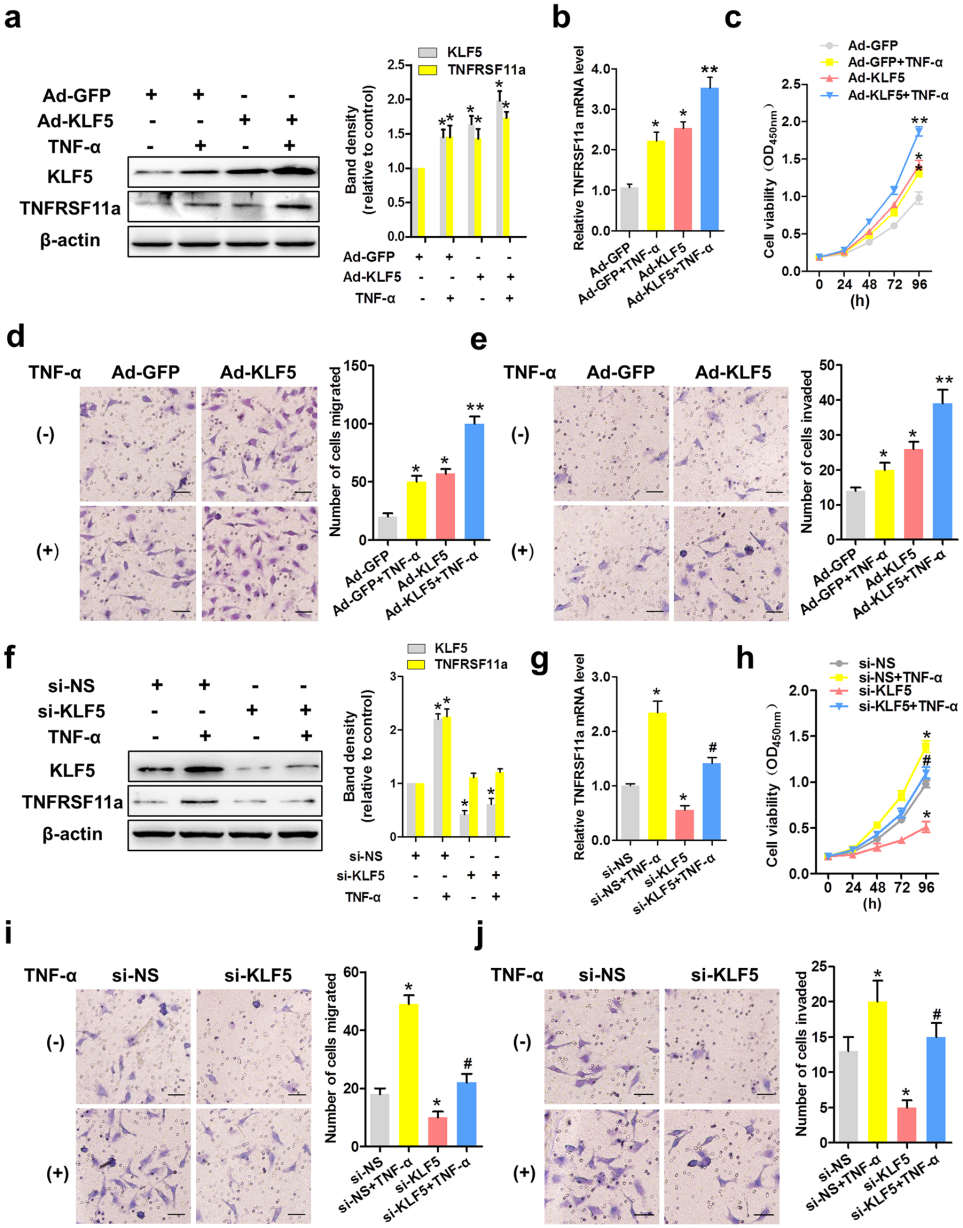
Krüppel-like factor 5 (KLF5) mediates tumour necrosis factor (TNF)-α-induced cervical cancer cell proliferation, migration and invasion. HeLa cells were infected with adenoviruses expressing green fluorescent protein (Ad-GFP) or KLF5 (Ad-KLF5) for 24 h and subsequently treated or not with TNF-α (10 ng/mL). KLF5 and TNFRSF11a protein and mRNA expression were analysed by western blotting (**a**) and qRT-PCR (**b**), respectively. *P < 0.05, **P < 0.01 vs. the Ad-GFP group. (**c**) Stable KLF5 overexpression in HeLa cells significantly promoted cell growth compared with stable GFP expression during a 96 h period, as measured by the CCK-8 assay. Data represent the means ± standard errors of the means (SEM; n = 3). *P < 0.05, **P < 0.01 vs. the Ad-GFP group. Representative photomicrographs of a Transwell assay using HeLa cells treated as described above. (**d**) and (**e**) present quantified analyses of cell migration and invasion, respectively. Data represent the means ± SEM (n = 3). *P < 0.05, **P < 0.01 vs. the Ad-GFP group. HeLa cells were transfected with non-specific short interfering RNA (siRNA; si-NS) or KLF5-specific siRNA (si-KLF5) for 24 h and subsequently treated or not with TNF-α. KLF5 and TNFRSF11a protein and mRNA expression were analysed by western blotting (**f**) and qRT-PCR (**g**), respectively. *P < 0.05 vs. the si-NS group, ^#^P < 0.05 vs. the TNF-α group. (**h**) Stable knockdown of KLF5 in HeLa cells significantly inhibited cell growth relative to the si-NS group during a 96-h period, as measured by the CCK-8 assay. Data represent the means ± SEM (n = 3). *P < 0.05 vs. the si-NS group, #P < 0.05 vs. the TNF-α group. In (**i**) and (**j**), representative photomicrographs of a Transwell assay show the respective quantification of HeLa cell migration and invasion after the above-described treatments. Data represent the means ± SEM (n = 3). *P < 0.05 vs. the si-NS group, #P < 0.05 vs. the TNF-α group.

KLF5 has been reported to exert pro-oncogenic activity by regulating gene transcription and stimulating cancer cell progression^10^. TNF-α is an indispensable cytokine that regulates the local microenvironment and thereby promotes CC progression. To further investigate whether KLF5 is an effector of TNFRSF11a and whether KLF5 plays a role in TNF-α-induced CC cell functions, we overexpressed KLF5 in HeLa cells through infection with a specific adenovirus (Ad-KLF5). As shown in Fig. 2a and b, KLF5 overexpression led to consistent upregulation of TNFRSF11a protein and mRNA in HeLa cells. We further tested HeLa cell proliferation using a CCK-8 assay and migration and invasion using a Transwell assay. As shown in Fig. 2c, TNF-α significantly induced the proliferation of HeLa cells. Additionally, treatment with TNF-α and/or Ad-KLF5 led to increased migration and invasion in a Transwell assay and thereby indicated an increased metastatic potential, compared to HeLa cells expressing only green fluorescent protein (Ad-GFP) (Fig. 2d,e). We also used short interfering RNA (siRNA) to inhibit the expression of KLF5 in HeLa cells and further investigate its biological function. As expected, KLF5 knockdown dramatically reduced both TNFRSF11a protein and mRNA expression in HeLa cells (Fig. 2f,g) and the CCK-8 assay indicated that the knockdown of KLF5 inhibited the proliferation of HeLa cells, regardless whether TNF-α was present or absent (Fig. 2h). Moreover, KLF5 siRNA-treated HeLa cells exhibited significantly suppressed TNF-α-induced migration (Fig. 2i) and invasion (Fig. 2j), compared with control-treated cells. Collectively, these results suggest that KLF5 affects TNFRSF11a expression and thereby promotes TNF-α-induced proliferation, migration and invasion in CC cells.

### TNFRSF11a mediates the proliferation, migration and invasion of CC cells

**Figure 3:**
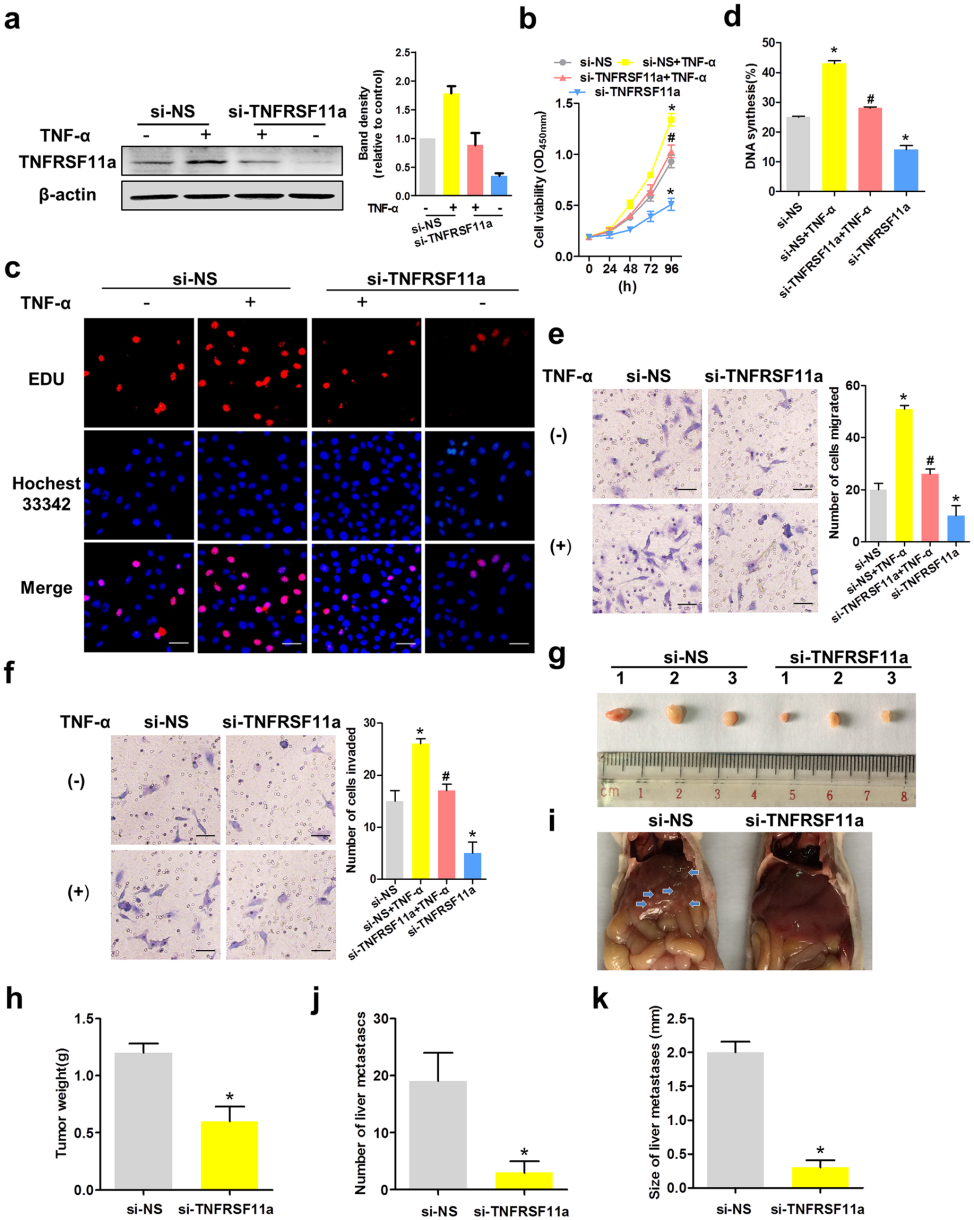
Tumour necrosis factor (TNF)-α induced TNFRSF11a-mediated cervical cancer cell proliferation, migration and invasion. (**a**) HeLa cells were transfected with non-specific short interfering RNA (siRNA; si-NS) or TNFRSF11a-specific siRNA (si-TNFRSF11a) for 24 h and subsequently treated or not with TNF-α. TNFRSF11a protein expression was analysed by western blotting. β-actin was used as the loading control. (**b**) Stable knockdown of TNFRSF11a significantly inhibited HeLa cell growth compared to treatment with si-NS during a 96-h period, as measured by a CCK-8 assay. Data represent the means ± standard errors of the means (SEM; n = 3). *P < 0.05 vs. the si-NS group, ^#^P < 0.05 vs. the TNF-α group. (**c**) Proliferating HeLa cells were labelled with EdU, which was revealed using the Click-it reaction (red). Cell nuclei were stained with Hoechst 33342 (blue). Representative images are shown. Scale bars = 50 μm. (**d**) Quantitative results from panel c. Data represent the means ± SEM (n = 3). *P < 0.05 vs. the si-NS group, ^#^P < 0.05 vs. the TNF-α group. TNFRSF11a knockdown altered HeLa cell migration (**e**) and invasion (**f**) in Transwell assays. Data represent the means ± SEM (n = 3). *P < 0.05 vs. the si-NS group, ^#^P < 0.05 vs. the TNF-α group. (**g**) HeLa-si-NS and HeLa-si-TNFRSF11a xenograft tumour masses were harvested on day 28. (h) TNFRSF11a knockdown significantly decreased the xenograft tumour weights, compared with si-NS tumours. *P < 0.05 vs. the si-NS group. (**i**) Photographs of livers removed from mice in each group. (**j**) Significantly fewer liver metastases were detected in the si-TNFRSF11a group, compared with the si-NS group. *P < 0.05 vs. the si-NS group. (**k**) Calculated average sizes of liver metastases removed from the mice in each group. *P < 0.05 vs. the si-NS group.

TNFRSF11a was previously shown to promote migration and invasiveness in breast and prostate cancers^29,30^. To determine whether this molecule plays a similar function in CC, we sought to measure HeLa and SiHa cell proliferation, migration and invasion after manipulating TNFRSF11a expression. We used human TNFRSF11a-specific siRNA (si-TNFRSF11a) or non-specific siRNA (si-NS) to knock down endogenous TNFRSF11a in HeLa and SiHa cells, and demonstrated that in response to TNF-α, TNFRSF11a expression was markedly attenuated in si-TNFRSF11a-transfected HeLa (Fig. 3a) and SiHa (Fig. S3a) cells relative to cells transfected with si-NS, and cell growth was significantly decreased in the former (Fig. 3b and Fig. S3c). As shown in Fig. 3c and d, the frequency of 5-ethynyl-2′-deoxyuridine (EdU)-positive HeLa cells decreased significantly under conditions of TNFRSF11a knockdown. As shown in Fig. 3e–f and Fig. S3d,e, treatment with si-TNFRSF11a led to significantly decreased HeLa and SiHa cell migration and invasion, compared to treatment with si-NS. Collectively, these results suggest that TNFRSF11a promotes the proliferation, migration and invasion of CC cells.

Next, we evaluated the effects of endogenous TNFRAF11a expression on the proliferative and metastatic potential of HeLa cells *in vivo*. Accordingly, we subcutaneously injected HeLa-si-NS and HeLa-si-TNFRSF11a cells into severe combined immunodeficiency (SCID) mice. In this model, TNFRSF11a knockdown significantly suppressed HeLa tumour growth, leading to lower mean tumour weights and few liver metastases after 28 days (Fig. 3g–k).

### TNF-α induced KLF5 positively regulates TNFRSF11a expression by binding to the TNFRSF11a promoter

**Figure 4:**
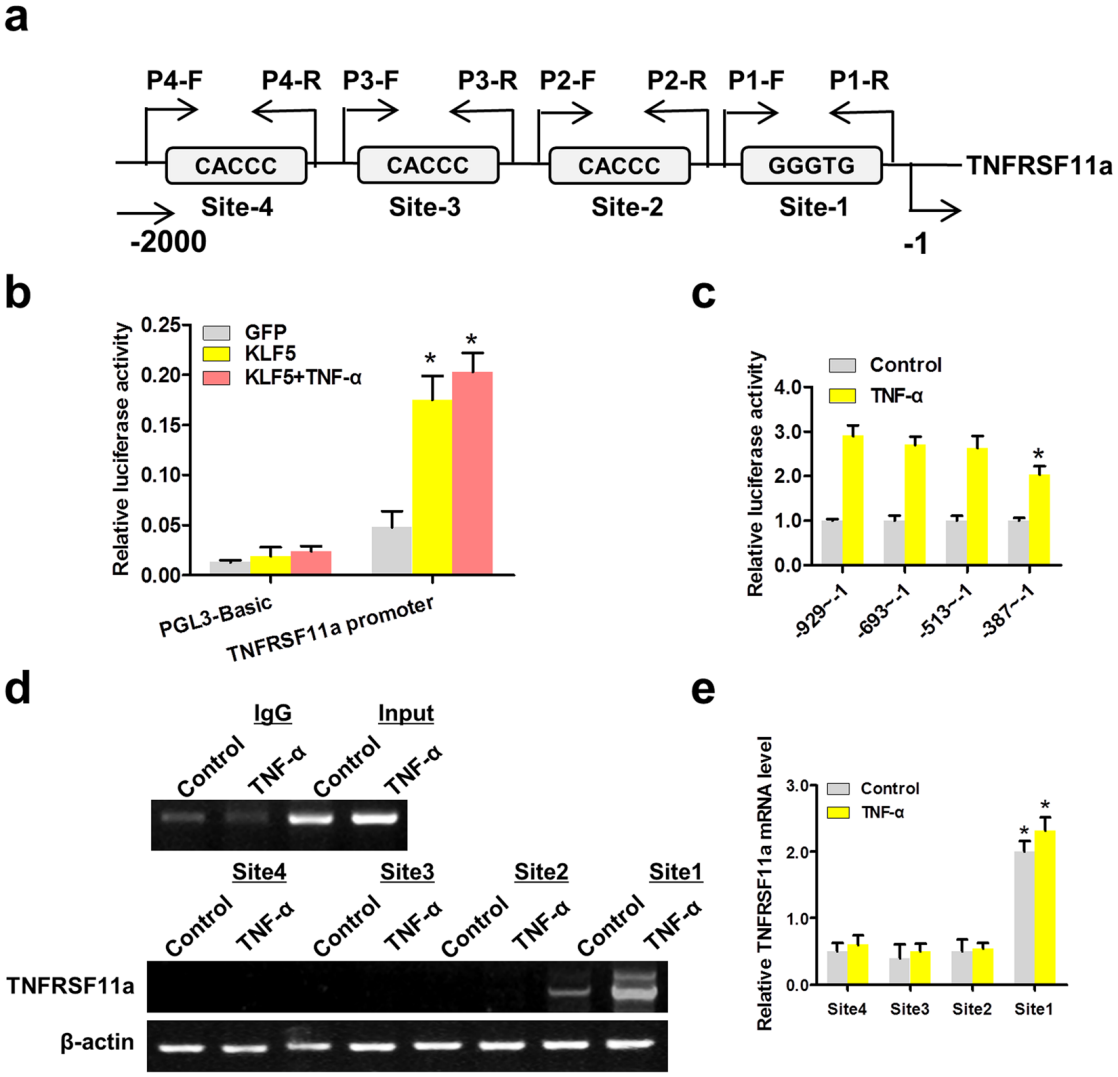
KLF5expression induced by tumour necrosis factor (TNF)-α, positively regulates *TNFRSF11a* expression by binding to the *TNFRSF11a* promoter. (**a**) Schematic map of the *TNFRSF11a* promoter region from −2000 to −1, with the positions of KLF5-binding sites. The arrows represent polymerase chain reaction (PCR) primers used in the chromatin immunoprecipitation (CHIP) assay. (**b**) KLF5 significantly activated the *TNFRSF11a* promoter in HeLa cells, as shown using dual-luciferase assays. KLF5 was overexpressed via infection with an adenovirus construct. pGL3-Basic was used as the negative control. The β-actin promoter-driven Renilla luciferase gene (pRL-β-actin) was used as the internal control. *P < 0.05. (**c**) HeLa cells were transfected with *TNFRSF11a* promoter-reporter plasmids containing various 5′-deletion fragments. Luciferase activity was measured as described above. *P < 0.05 vs. promoter-reporters containing the −929 to −513 region. (**d**,**e**) HeLa cells were incubated with or without TNF-α and whole-cell lysates were subjected to CHIP assays using the positive KLF5-binding site sequences (sites 1–4) as probes. DNA-bound proteins were collected with streptavidin-agarose beads and analysed by western blotting with an anti-KLF5 antibody. *P < 0.05.

Because both KLF5 overexpression and knockdown altered *TNFRSF11a* expression individually, we further identified the molecular mechanisms of regulation of TNFRSF11a expression by transcription factor KLF5. Using a TESS-String-based Search (//www.cbil.upenn.edu/tess/), we found that the 2000/+1 bp region of the *TNFRSF11a* promoter contained four KLF5-binding sites (Fig. 4a). To investigate whether KLF5 activated the transcription of *TNFRSF11a*, we co-transfected HeLa cells with a KLF5 expression plasmid and *TNFRSF11a* promoter–luciferase reporter (pGL3-TNFRSF11a-Luc) plasmid in the presence or absence of TNF-α treatment. A luciferase assay demonstrated significant activation of the *TNFRSF11a* promoter by KLF5 (Fig. 4b). Another luciferase assay was conducted to determine the KLF5-binding sites in TNFRSF11a promoter regions (Fig. 4c). The results showed that TNF-α could partly promote the binding of KLF5 to the proximal region of the *TNFRSF11a* promoter (−387 bp to 1 bp), which contains KLF5-binding site 1; in contrast, no significant binding of KLF5 was detected when the distal *TNFRSF11a* promoter region containing KLF5-binding sites 2–4 was amplified. Consistent with the results of the luciferase assay, chromatin immunoprecipitation (CHIP) assays demonstrated significantly increased binding of KLF5 to site 1 (Fig. 4d,e). These results indicate that TNF-α induces the binding of KLF5 to the proximal region of the *TNFRSF11a* promoter in HeLa cells, thus increasing *TNFRSF11a* transcription.

### KLF5 promotes HeLa cell proliferation and migration in a manner partly dependent on TNFRSF11a expression

**Figure 5:**
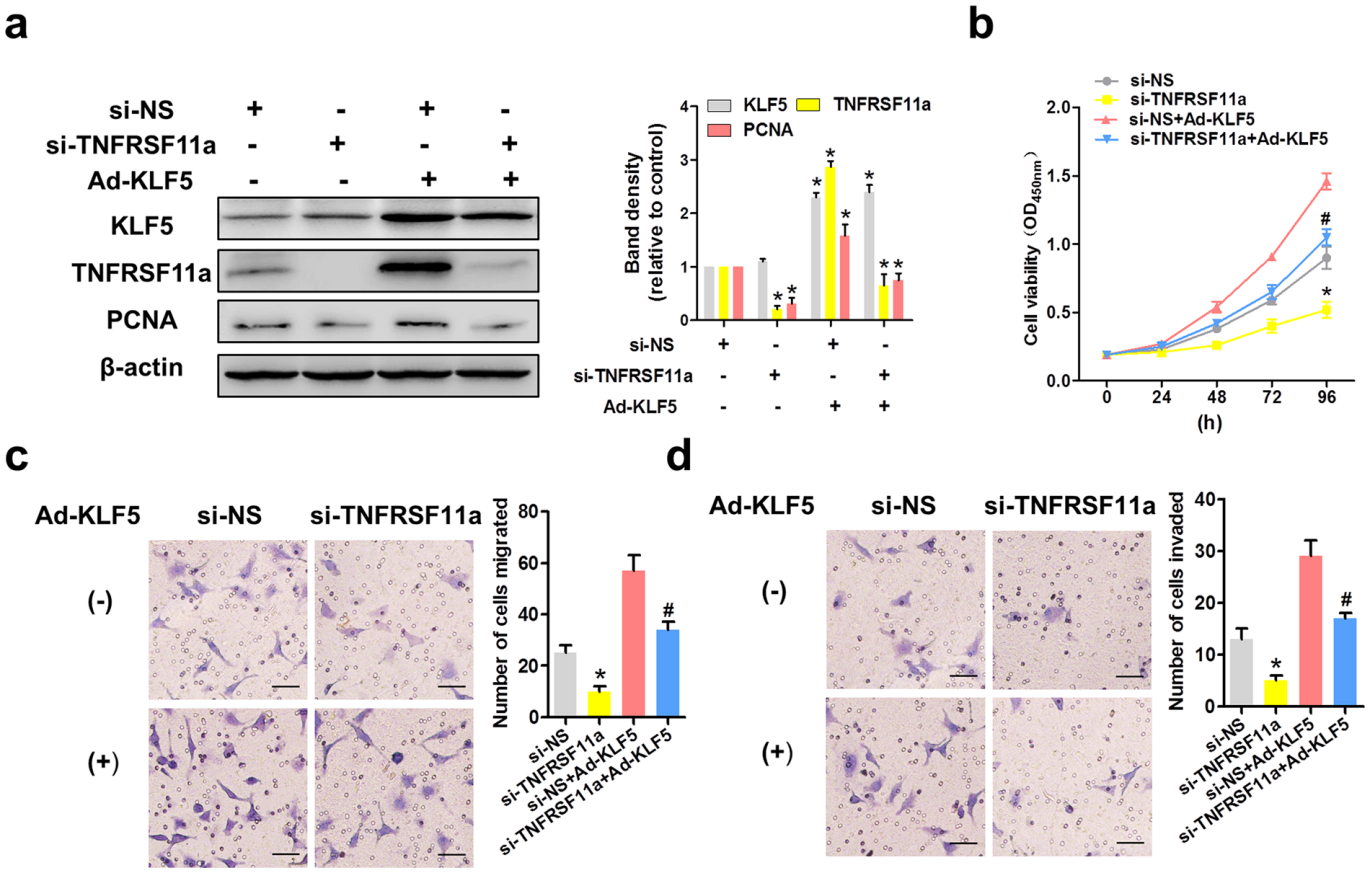
KLF5 promotes HeLa cell proliferation and migration in a manner partly dependent on tumour necrosis factor receptor superfamily member 11a (TNFRSF11a) expression. (**a**) TNFRSF11a knockdown and KLF5 overexpression in HeLa cells were confirmed by western blotting. β-actin was used as the loading control. (**b**) TNFRSF11a knockdown and KLF5 overexpression altered HeLa cell growth relative to the control during a 96 h period, as measured by a CCK-8 assay. Data represent the means ± standard errors of the means (SEM; n = 3). *P < 0.05 vs. the si-NS group, #P < 0.05 vs. the Ad-KLF5 group. TNFRSF11a knockdown and KLF5 overexpression also affected HeLa cell migration (**c**) and invasion (**d**), as shown by Transwell assays. Data represent the means ± SEM (n = 3). *P < 0.05 vs. the si-NS group, #P < 0.05 vs. the Ad-KLF5 group.

To determine whether KLF5 activity depends on TNFRSF11a expression, we simultaneously overexpressed KLF5 and knocked down TNFRSF11a in HeLa cells (Fig. 5a). As shown in Fig. 5b, KLF5 overexpression promoted the proliferation of si-NS-transfected HeLa cells, whereas this effect was attenuated in TNFRSF11a-knockdown cells. In addition, KLF5 overexpression could not totally rescue migration and invasion in TNFRSF11a-knockdown HeLa cells (Transwell assays; Fig. 5c,d). Overall, these data suggest that KLF5 promotes proliferation, migration and invasion in HeLa cells, partially by inducing TNFRSF11a expression.

### TNF-α induces KLF5 expression via the p38 pathway in CC cells

**Figure 6:**
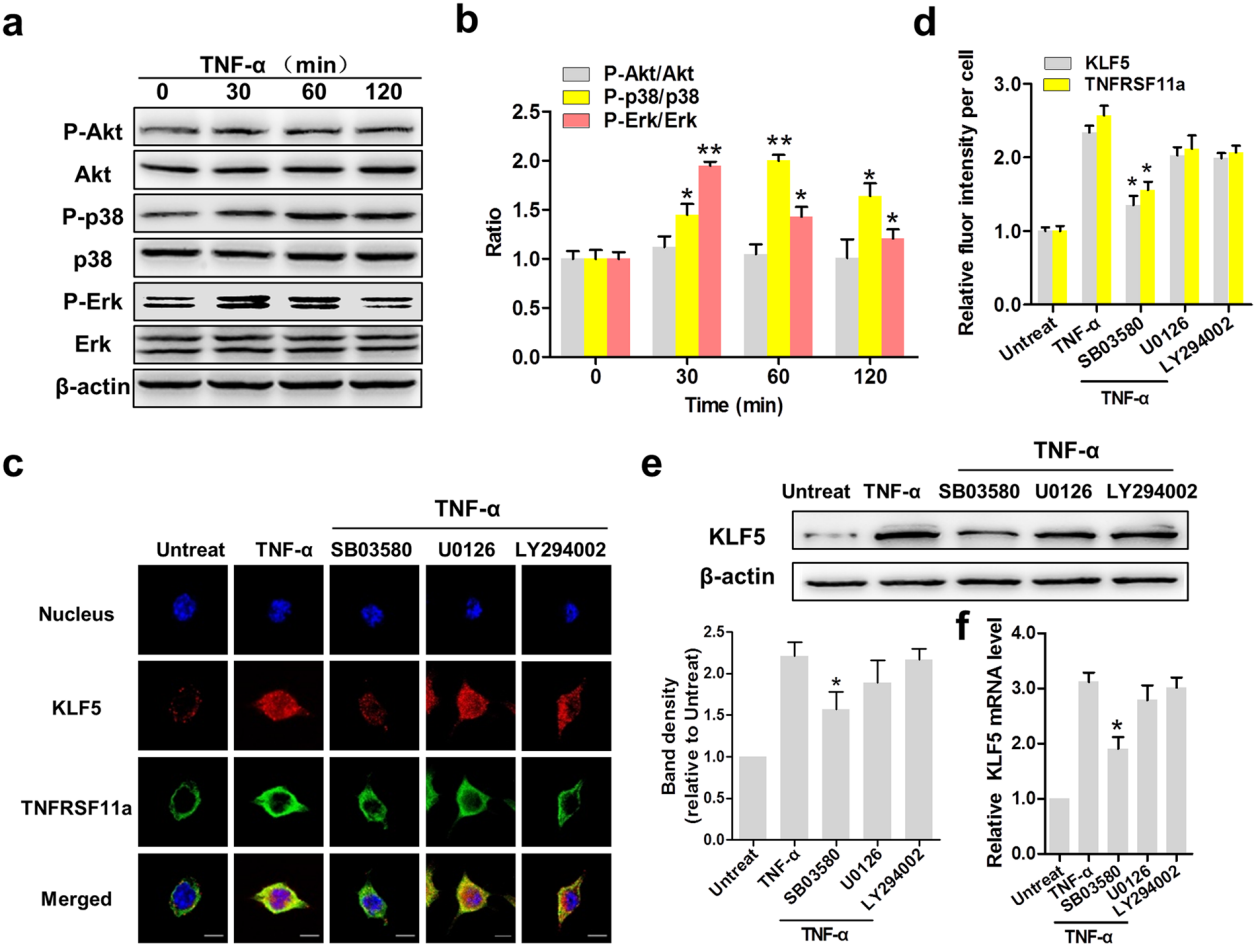
TNF-α induces KLF5 expression via the p38 pathway in cervical cancer cells. (**a**) Erk, Akt and p38 MAPK phosphorylation in HeLa cells was analysed by western blotting. β-actin was used as the loading control. (**b**) The activation levels of these three kinases were determined by the respective phosphorylation ratios. *P < 0.05, **P < 0.01. (**c**) The cellular locations of KLF5 (red) and tumour necrosis factor receptor superfamily member 11a (TNFRSF11a, green) in HeLa cells were determined by immunofluorescence staining (×200). DAPI was used for nuclear staining. Scale bars = 10 μm. (**d**) Statistical analysis of fluorescence intensity. *P < 0.05 vs. the TNF-α group. KLF5 expression in HeLa cells was determined at the protein (**e**) and mRNA levels (**f**). *P < 0.05 vs. the TNF-α group.

Because Erk, AKT and p38 activation are required for cell proliferation and migration^32–34^, we sought to determine whether these kinases affected KLF5 expression in TNF-α-stimulated HeLa cells. After treatment for different intervals of time, Erk and p38 phosphorylation increased to maximum levels at 30 and 60 min, respectively, followed by gradual decreases within 120 min after treatment; in contrast, Akt phosphorylation did not appear to be affected (Fig. 6a,b). As shown in Fig. 6c, KLF5 localises to the cytoplasm in HeLa cells, whereas TNFRSF11a localises at the cell membrane. However, treatment with TNF-α increased the nuclear fraction of KLF5. Next, we treated HeLa cells with U0126 (Erk inhibitor), Ly294002 (Akt inhibitor) and SB03580 (p38 inhibitor). Notably, treatment with SB03580 led to a significant decrease in the nuclear fraction of KLF5, whereas Ly294002 and U0126 did not have evident effects (Fig. 6d, P < 0.05). A western blotting analysis indicated a significant decrease in KLF5 protein expression in SB03580-treated HeLa cells, compared to TNF-α-treated cells (Fig. 6e). A qRT-PCR analysis also indicated significant decreases in KLF5 mRNA levels (Fig. 6f, P < 0.05). These findings suggest that TNF-α induces KLF5 expression in CC cells via the p38 pathway.

### KLF5 and TNFRSF11a expression in cervical squamous cell carcinoma tissues correlates with patient survival

**Figure 7:**
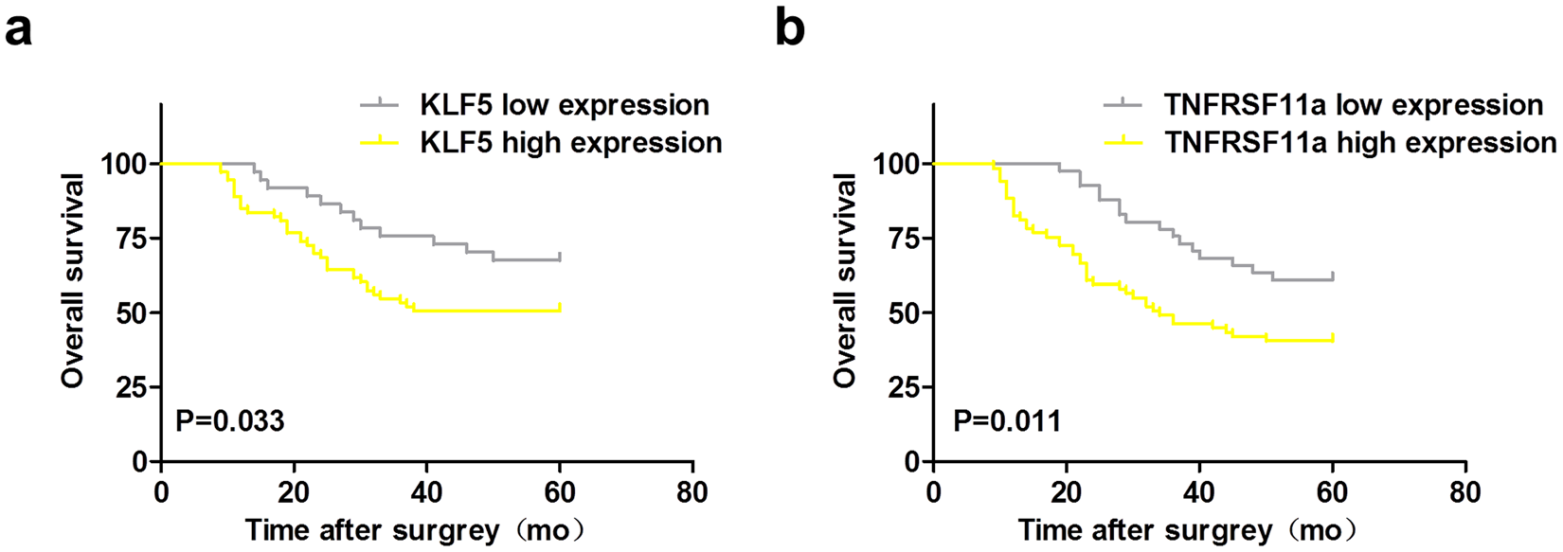
KLF5 and TNFRSF11a expression in cervical squamous cell carcinoma (CSCC) tissues correlates with patient survival. (**a**) Patients were divided into two groups, low or high KLF5 expression, based on the minimum observed P value. The OS of patients with low and high KLF5 expression was evaluated using a Kaplan–Meier survival analysis (log-rank P = 0.033). (**b**) Patients were also divided into two groups, low or high TNFRSF11a expression, based on the minimum observed P value. The OS of patients with low and high TNFRSF11a expression was evaluated using a Kaplan–Meier survival analysis (log-rank P = 0.011).

Table 1 lists the characteristics of 110 participants, with a median age of 45 years (range: 25–79 years). Significant differences in tumour size, histological type, invasion depth, nodal metastasis status, pathological grade, Union for International Cancer Control (UICC) stage and disease-free survival outcomes were detected between the two KLF5 groups and TNFRSF11a groups (P < 0.05). No significant inter-group differences in other clinical variables were observed (P > 0.05). Figure 7a presents overall survival (OS) curves for the low and high KLF5 expression groups. Patients in the latter group had a significantly lower OS, compared with those in the former group (log-rank, P = 0.033 for OS). Figure 7b presents the OS curves for the low and high TNFRSF11a expression groups. Again, patients in the latter group had a significantly lower OS, compared with those in the former group (log-rank, P = 0.011 for OS).

**Table 1:** Baseline characteristics of the low and high KLF5 and TNFRSF11a expression groups (n = 110).

Clinicopathological characterisitics	No.(%)	KLF5 expression High No.(%) Low No. (%)		*P*	TNFRSF11a expression High No. (%) Low No. (%)		*P*
Age(year)							
≤45	41(37.27%)	24(32.88%)	17(45.95%)	0.180	29(42.03%)	12(29.27%)	0.181
>45	69(62.73%)	49(67.12%)	20(54.05%)		40(57.97%)	29(70.73%)	
Histological type							
High, Middle differentiatiated	72(65.46%)	42(57.53%)	30(81.08%)	0.014*	37(53.62%)	35(85.37%)	0.001*
Poorly differentiated	38(34.55%)	31(42.47%)	7(18.92%)		32(46.38%)	6(14.63%)	
Tumor size (m^2^)							
≤3	66(60.00%)	46(63.01%)	20(54.05%)	0.365	40(57.97%)	26(63.41%)	0.573
>3	44(40.00%)	27(36.99%)	17(45.95%)		29 (42.03%)	15(36.59%)	
Infiltration depth							
≤1/2	75(68.18%)	55(75.34%)	20(54.05%)	0.024*	52(75.36%)	23(56.10%)	0.036*
>1/2	35(31.82%)	18(24.66%)	17(45.96%)		17(24.64%)	18(43.90%)	
Nodal metastasis							
N0	64(58.18%)	36(49.32%)	28(75.68%)	0.008*	33(47.83%)	31(75.61%)	0.004*
N1	46(41.82%)	37(50.69%)	9(24.32%)		36(52.17%)	10(24.39%)	
FIGO stage							
I	61(55.45%)	46(63.01%)	15(40.54%)	0.025*	44(63.77%)	17(41.46%)	0.023*
II	49(44.55%)	27(36.99%)	22 (59.46%)		25(36.23%)	24 (58.54%)	

Asterisk denotes significant *P* values (*P* < 0.05).

## Discussion

KLF5 functions as an oncogenic transcription factor in several cancer types, including breast^22^, bladder^35^ and intestinal cancers^36^. According to research conducted by Marrero, the expression of KLF5 mRNA gradually increases in CC cells^37^. In this study, we identified *TNFRSF11a* as a direct target of KLF5 and, most importantly, demonstrated that KLF5 promoted the proliferation, migration and invasion in HeLa cells by inducing TNFRSF11a expression. We initially observed KLF5 and TNFRSF11a mRNA and protein coexpression in cervical tumours, and identified KLF5 as an effector of TNFRSF11a. Furthermore, we showed that KLF5 mediated TNF-α-induced proliferation, migration and invasion in CC cells, and bound to the *TNFRSF11a* gene promoter to increase transcription activity and promotes *TNFRSF11a* expression in HeLa cells. Finally, we demonstrated that TNFRSF11a mediates CC cell proliferation, migration and invasion and that KLF5 promotes these processes in HeLa cells in a manner partly dependant on TNFRSF11a expression.

KLF5 expression can be induced by various oncogenic and pro-inflammatory factors, including TNF-α^38^, lipopolysaccharide^39^ and interleukin-1β^37^. TNF-α can also induce TNFRSF11a expression and, according to the present study, KLF5 expression. These findings imply that the KLF5/TNFRSF11a axis is responsive to various extracellular oncogenic and pro-inflammatory stimuli. Moreover, in osteoclasts, the TNFRSF11a (RANK)/RANK ligand (L) interaction activates a signalling cascade involving downstream targets such as the Erk, p38, Akt and nuclear factor (NF)-kB signalling pathways^40,41^. KLF5 has also been shown to regulate target genes by interacting with NF-κB^42^. Further investigation is needed to determine whether KLF5 and NF-κB form a transcription complex at the *TNFRSF11a* promoter and co-ordinately regulate *TNFRSF11a* transcription in CC cells.

Previously, KLF5 was reported to promote breast cancer proliferation, migration and invasion in part by upregulating the transcription of TNFAIP2^43^. In this study, we demonstrated that KLF5 promoted HeLa cell proliferation, migration and invasion in a manner at least partly mediated by TNFRSF11a. However, we could not exclude the possibility that other KLF5 target genes might also contribute to HeLa cell migration and invasion. Further studies to determine whether KLF5 and TNFRSF11a promote CC metastasis *in vivo* are expected to yield significant results.

TNFRSF11a expression has been reported in osteogenic sarcoma^44^ and in two types of tumours with high bone metastatic potential (breast and prostate cancer)^30,31^, but has not been well investigated in CC. This study is the first to demonstrate that TNFRSF11a depletion suppresses CC cell proliferation *in vitro* (Fig. 4), thus suggesting that TNFRSF11a could serve as a therapeutic target in CC. Interestingly, TNFRSF11a also promoted HeLa cell migration and invasion.

We next explored the potential functional mechanism underlying the observed effects. In previous studies, KLF5 promoted breast cell survival partly through fibroblast growth factor-binding protein 1-pERK-mediated dual specificity MKP-1 protein phosphorylation and stabilization^32^ and could activate MEK/Erk signalling and cell proliferation^45^. Moreover, TNFRSF11a was reported to activate signalling pathways downstream of its ligand RANKL, such as NF-κB, JNK, Erk, p38 and Akt/PKB, via TRAF protein phosphorylation^41,46,47^. In our experiments, TNF-α induced KLF5 expression in CC cells via the p38, but not the Erk or Akt, pathway.

Besides our more conclusive findings regarding the role of the KLF5/TNFRSF11a in CC cells, our results offer more suggestive and will require additional, more targeted follow-up. Meanwhile, the clinical studies suggest an association of KLF5 mRNA expression with metastasis and recurrence in patients with CC (Table 1), consistent with the idea that KLF5 promotes cell proliferation and migration. Although these results may be significant, they are rather generalised and further study is needed to confirm the expression of KLF5 and TNFRSF11a mRNA and protein in clinical CC tumours.

In recent years, an explosion of studies has led researchers to decode the substrates of KLF5 that affect the growth, migration and invasion of CC cells. Delineation of the affected CC tissues and conditions under which KLF5 is active remains a critical goal of research aimed at understanding the effects of downstream targets on growth and migration. A much more comprehensive analysis of KLF5 and its targets, including analyses of genetic losses of function and RNA interference, is needed to determine the relative importance of KLF5 in CC cell lines and cervical neoplasia.

In conclusion, we have demonstrated the coexpression of KLF5 and TNFRSF11a in CC. We found that at a genetic level, KLF5 tightly controls *TNFRSF11a* transcription by binding to the *TNFRSF11a* promoter and in response to TNF-α activation, KLF5 promotes HeLa cell proliferation, migration and invasion partly through TNFRSF11a. These findings suggest that KLF5 and TNFRSF11a may be valuable therapeutic targets in CC.

## Methods

### Clinical samples

Human tissue samples were obtained from 218 patients (40 normal cervical tissues, 68 cervical intraepithelial neoplasia (CIN) tissues (CIN I: 23 cases; CIN II-III: 45 cases) and 110 CSCC tissues) from January 2008 to December 2010. A total of 140 CSCC patients were enrolled; however, 30 patients were excluded due to lack of pathological specimen or other reason, loss to follow up at the early stage of the study, resulting in 110 participants in the final study population. The median age of all the participants was 45 years, and the age range was 25 to 79 years. All of these patients have been informed consent before collection of their samples, according to institutional guidelines. This protocol was approved by the Ethics Committee of Hebei United University (No. 15007), in Tangshan of Hebei province, China. After diagnosis, they underwent surgical resection of primary cervical cancer at the Department of Obstetrics and Gynecology in Tangshan workers hospital. The histological type and grade of tumor were classified on the basis of WHO criteria. The stage of each cancer was established according to the International Federation of Gynaecology and Obstetrics (FIGO) criteria. These tissue samples for CIN diagnosis were performed by using micro-excision. All primary tumor tissues and control samples were diagnosed by HE-stained. A part of the samples were paraffin embedded for Immunehistochemical staining, and another part is within the sterilization of the aluminum foil and preserved in liquid nitrogen for qRT-PCR.

### Study endpoints

Primary endpoints were overall survival (OS). OS was defined as the time from registration until death from any cause. Surviving patients were censored on March 30, 2016. The Ethics Committee of Hebei United University approved this study (Approval No. 15007), which conformed to the requirements of the Declaration of Tangshan, and all patients gave informed consent prior to all procedures. All methods were carried out in accordance with relevant guidelines and regulations. All experimental protocols were approved by the licensing committee of North China University of Science and Technology.

### Cell culture

Human cervical (HeLa, SiHa and C33A) cancer cells were purchased from the Cell Culture Center (Manassas, VA). They were cultured in high glucose Dulbecco’s modified Eagle’s medium (DMEM) (GibcoLaboratories, Grand Island, NY, USA) containing 10% heat-inactivatedfetal bovine serum, 100 units/mL penicillin, and 100 μg/mL strepto-mycin (Invitrogen, Carlsbad, CA) at 37 °C in a 5% CO2/ 95% air humidified atmosphere. The medium was changed every two days during incubation.

### Double immunofluorescence staining

Immunofluorescence staining was performed with 5 μm paraffincross-sections from the human cervical tissues. After deparaffinized in xylene and rehydrated in graded alcohol, the slides were pre-incubated with 10% normal goat serum and then incubated with primary antibodies rabbit anti-KLF5 (1:50, GTX103289, GeneTex), mouse anti-TNFRSF11a (1:50, MBS245189, Mybiosource). Secondary antibodies were fluoresce in labeled antibody to mouse IgG (021815, KPL, USA), rhodamine labeled antibody to rabbit IgG (031506, KPL, USA). In each experiment, DAPI (0.1 ug/ml, 157574, MB biomedical) was used for nuclear counter staining. Images were captured by confocal microscopy (DM6000 CFS, Leica) and processed by LAS AF software.

### Plasmids DNA and transfection

Lipofectamine 2000 (Invitrogen, Carlsbad, CA, USA) was used for all transfections with siRNA and plasmids, according to the manufacturer’s instructions. After selection, western blotting was performed to confirm if the knockdown was effective. Preparation of Plasmid Constructs and Recombinant adenoviral-KLF5 constructs were constructed as described previously12. The transfected cells were cultured in complete medium for 16 h and then, in normal growth medium for additional 48 h before performing functional assays or harvesting cell lysates for protein expression analyses.

### Transient transfection of HeLa and SiHa cells

HeLa and SiHa cells were infected with Ad-KLF5 or Ad-GFP as described previously [13]. Non-specific siRNA (NS siRNA) was purchased from Santa Cruz Biotechnology. The siRNA sequences used in these studies were as follows: KLF5 siRNA: 5′-CGAUUACCCUGGUUGCACA-3′ and 5′-AAGCU- CACCUGAGGACUCA-3′; TNFRSF11a siRNA: 5′-GACUUGGGCUCACAGAU- AA-3′ and 5′-GAUUGAGGUGGCCACUUAU-3′; Luciferase siRNA/shRNA, 5′-CUUACGCUGAGUACUUCGA-3′. Transfection was performed using Lipofectamine TM reagent (Invitrogen) according to the manufacturer’s instructions. 24 h after transfection, HeLa and SiHa cells were treated with TNF-α (10 ng/mL). Then cells were harvested and lysed for Western blotting.

### qRT-PCR

Total mRNA from cervical tissues or HeLa cells were extracted with the RNeasyplus mini kit (QIAGEN, Valencia, CA, USA) according to the manufacturer’s instructions, and were reverse transcribed into cDNA (TaKaRa Bio Inc., Tokyo,Japan). The cDNA was then amplified by real-time quantitative TaqMan PCR with KLF5 F, 5′-ACACCAGACCGCAGCTCCA-3′ and KLF5 R, 5′-TCCATTGCTGCTGTCTGATTTGTAG-3′; TNFRSF11a F, 5′-TTTCCGGGA- GGAGCTCATGG-3′ and TNFRSF11a R,5′-CAGGTGGCCTTTGCTGAAGT-3′; β-actin F,5′-GGTGAAGGTCGGAGTCAACG-3′ and β-actin R, 5′-TGGGTGGA- ATCATATTGGAACA-3′, specific primers and probes with the Lightcycler 480 II (Roche, Mannheim, Germany). The data were normalized to β-actin and expressed as the fold change over control. The relative expression level was calculated using the following equation: relative gene expression = 2^−(△CTsample−△CTcontrol)^.

### Western blotting

Total proteins from HeLa cells were extracted with cell lysis reagent (Promega, Madison, WI, USA), and supplemented with Complete Mini protease in hibitorcocktail (Roche Diagnostics, GmbH, Germany). The protein samples were loaded on and separated by SDS-PAGE, after being quantitatively determined by using Bradford Reagent (Bio-Rad, Hercules, CA, USA), and then were transferred to PVDF membranes, which were then blocked in 5% skimmed milk for 1 h at room temperature and probed with an antibody to KLF5, TNFRSF11a, PCNA, Akt, P-Akt, p38, P-p38, Erk, P-Erk or β-actin (Santa Cruz Biotechnology, SantaCruz, CA, USA). Antibody binding was detected by using chemiluminescence (Thermo Scientific, Rockford,USA) according to the manufacturer’s instructions with a peroxidase-conjugated anti-mouse antibody. The housekeeping gene β-actin was used as an internal control. The data were expressed as percentage to β-actin.

### Dual luciferase assays

The proximal TNFRSF11a promoters were amplified using normal human DNA as a template and cloned into the pGL3-Basic (Promega) vector. HeLa cells were seeded in 24-well plates at 1 × 105 cells/well. On the next day, the cells were transfected with the TNFRSF11a reporter plasmid (0.6 μg/well) and a pRL-β-actin internal control (5 ng/well) in triplicate. At 24 h after transfection, the cells were separately infected with a green fluorescent protein (GFP) control adenovirus and a KLF5 adenovirus for 4 h (∼50% cells were infected under a fluorescence microscope). At 48 h after transfection, luciferase activity was measured using the dual-luciferase reporter assay system (Promega).

### Chromatin immunoprecipitation assays

The chromatin immunoprecipitation assay was performed using the HeLa cells following the protocol provided by Abcam (Cambridge, MA, USA). The diluted DNA- protein complex was incubated with an equal amount of anti-TNFRSF11a antibody or mouse IgG (Santa Cruz) overnight at 4 °C in the presence of herring sperm DNA and protein A/G beads. Chromosomal DNA was purified and analyzed by RT-qPCR. The PCR primers for the TNFRSF11a gene promoter to amplify the region of interest were as follows: P1-F, 5′-GGCCAGTCTCCCGTCAGTCC-3′; P1-R, 5′-CTTTGCTATCTGGCGCTGGG-3′; P2-F, 5′-CCCTCTACCCACTGA- AGCGATA-3′; P2-R, 5′-GGAACGCCCCCAATACCTGC-3′; P3-F, 5′-GCAGC- GCAGTAGGGAAACAG-3′; P3-R, 5′-GAGCTTATCCCGGTCAGGCC-3′; P4-F, 5′-TTCCCAGATCCAGGCAAATGC-3′; P4-R, 5′- AGTCGTGCTGTCGCTAGG- CC-3′.

### Cell proliferation assay

Cell proliferation was estimated using a CCK-8 assay. HeLa cells were used for the logarithmic growth phase. Cell suspensions (8000 cells/well) were added to 96-well plates in a volume of 200 ml/well. Each group was prepared with five parallel wells and incubated at 37 °C, 5% CO2, for 48 h. At the end of the culture period, 10 ml CCK-8 (DOJINDO, Kumamoto, Japan) was added to each well. After 4 h incubation, the absorbance was measured with an enzyme calibrator at 450 nm after visual color occurrence at 24, 48, 72 or 96 h and the optical density (OD) values were measured. Experiments were repeated three times.

### EdU proliferation assay

5-ethynyl-2′-deoxyuridine (EdU) is a nucleoside analog of thymidine whose incorporation can be used to label cells undergoing DNA replication. Proliferating HeLa cells were evaluated by using the Click-iT EdU Alexa Fluor 594 Imaging Kit (Invitrogen, Carlsbad, CA, USA) according to the manufacturers’ instructions. Briefly, HeLa cells were incubated with 10 μM EdU for 3 h at 37 °C, fixed with 3.7% formaldehyde for 15 min, and treated with 0.5% Triton X-100 for 20 min at room temperature. After washing twice with PBS containing 3% BSA, the cells were reacted with Click-iT reaction cocktail for 30 min. Subsequently, cell nuclei were stained with Hoechst 33342 (Invitrogen, Carlsbad, CA, USA) at a concentration of 5 μg/mL for 30 min. The images were acquired by fluorescence microscopy and overlapped using Image-Pro Plus (Version 6.0.0.260, Media Cybernetics, Inc., Tokyo, Japan).

### *In vitro* migration and invasion assay

An *in vitro* migration and invasion assay was performed using a 48-well Boyden chamber as previously described. For knockdown KLF5 or TNFRSF11a assay, approximately 5 × 105 HeLa cells were added to the upper chamber in serum free media. The lower compartment was filed with serum-free media containing 10% FBS. For adenoviral infection KLF5 or transfection TNFRSF11a assay, approximately 1 × 105 HeLa cells were added to the upper chamber in serum free media. The lower compartment was filed with serum-free media containing 10% FBS. The assays were performed with or without Matrigel (BD Biosciences, San Jose, CA, USA), respectively. All cells were seeded in the upper part of the Boyden chamber and incubated for 12 h for migration and 24 h for invasion. These cells were fixed with 100% methanol and stained with 0.05% Giemsa for 30 mins. The migratory phenotypes were determined by counting the cells that migrated to the lower side of the filter by using microscopy at ×400.Thirteen fields were counted for each filter and each sample was assayed in triplicate.

### Animal Experiments

The HeLa cells population with stable TNFRSF11a knockdown was prepared by lentiviral transduction and puromycin selection. The effectiveness of the TNFRSF11a knockdown was confirmed by WB. A total of 20 6-week-old female SCID mice purchased from Vital River (Beijing, China) were randomly distributed into two groups (si-NS and si-TNFRSF11a, 10 mice per group, the technician just haphazardly assigned mice into different cages one by one as these mice are basically very similar in size and age). To produce subcutaneous tumors or experimental liver metastases, each mouse was injected subcutaneously with a mixture of tumor cells (1 × 10^7^ cells per site in 100 μl PBS). Mice were sacrificed 28 days after tumor implantation. Subcutaneous tumors were weighted and hepatic metastases were determined. All animal studies were approved by the Animal Experimental Ethical Inspection Form of Hebei United University (No. 2015-004) and all efforts were made to minimize suffering.

### Statistical analysis

Statistical analysis was performed using GraphPad Prism 5 software (GraphPad Software, La Jolla, CA). Sample size was chosen using the SPSS 17.0 statistical software to ensure adequate power to detect a prespecified effect size. All experiments were performed at least three times, and data are reported as the means ± SEM. The variance is similar between the groups that are being statistically compared. Differences between two given groups were analyzed by t-tests.

### Data availability statement

All data are fully available without restriction.

## Electronic supplementary material

Supplementary Information
