# Supplementary material for: KLF5 promotes cervical cancer proliferation, migration and invasion in a manner partly dependent on TNFRSF11a expression
Source: Sci Rep. 2017 Nov 16;7:15683. doi: 10.1038/s41598-017-15979-1 (PMC5691198; doi:10.1038/s41598-017-15979-1)
Supplement: Supplementary file 1 — Supplementary Information [file 41598_2017_15979_MOESM1_ESM.pdf]

KLF5 promotes cervical cancer proliferation, migration and invasion in a manner partly dependent on TNFRSF11a expression

Dong Ma [1], Ling-Ya Chang [1], Shan Zhao [2], Jun-Jian Zhao [3], Yan-Jie Xiong [3], Fu-Yuan Cao [1], Lu Yuan [1], Qi Zhang [1], Xin-Yue Wang [1], Mei-Li Geng [4], Huan-Yu Zheng [4] and Ou Li [4, *]

[1] School of Public Health, North China University of science and technology, Jianshe Road 57, Tangshan 063000, Hebei, China. [2] Department of Cancer Second Division, The Second Hospital of Hebei Medical University, 215 Heping West Road, Shijiazhuang 050000,Hebei, China. [3] North China University of Science and Technology Affiliated Hospital, Jianshe Road 57, Tangshan 063000, Hebei, China. [4] Department of Obstetrics and Gynecology, Workers' Hospital of Tangshan, Wenhua road 27, Tangshan 063000, Hebei, China. *Correspondence and requests for materials should be addressed to O.L. (email: tsliou@163.com).

Supplemental Figures

Figure S1

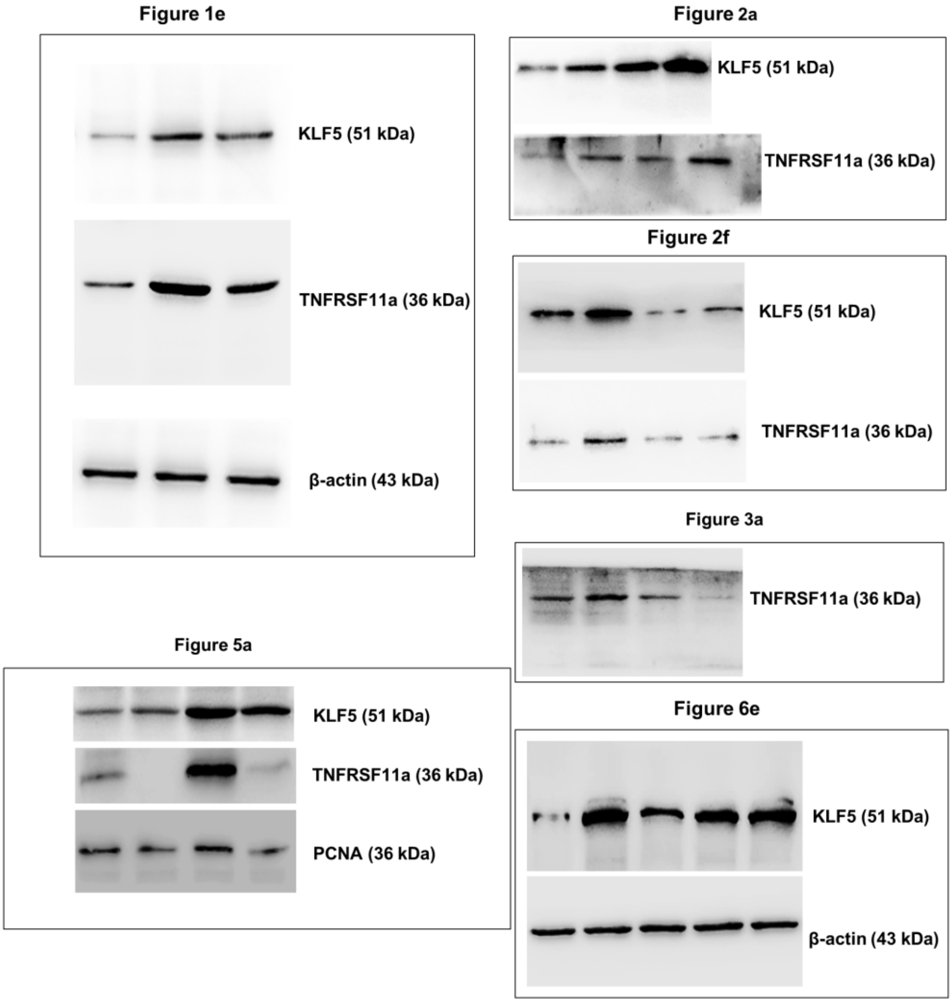

Fig. S1. Full-width of membrane with a protein marker besides the each box of the original blots used in Figures 1–6. The molecular size is shown as indicated. Panels shown in this supplemental figure correspond to those in the main article.

Figure S2

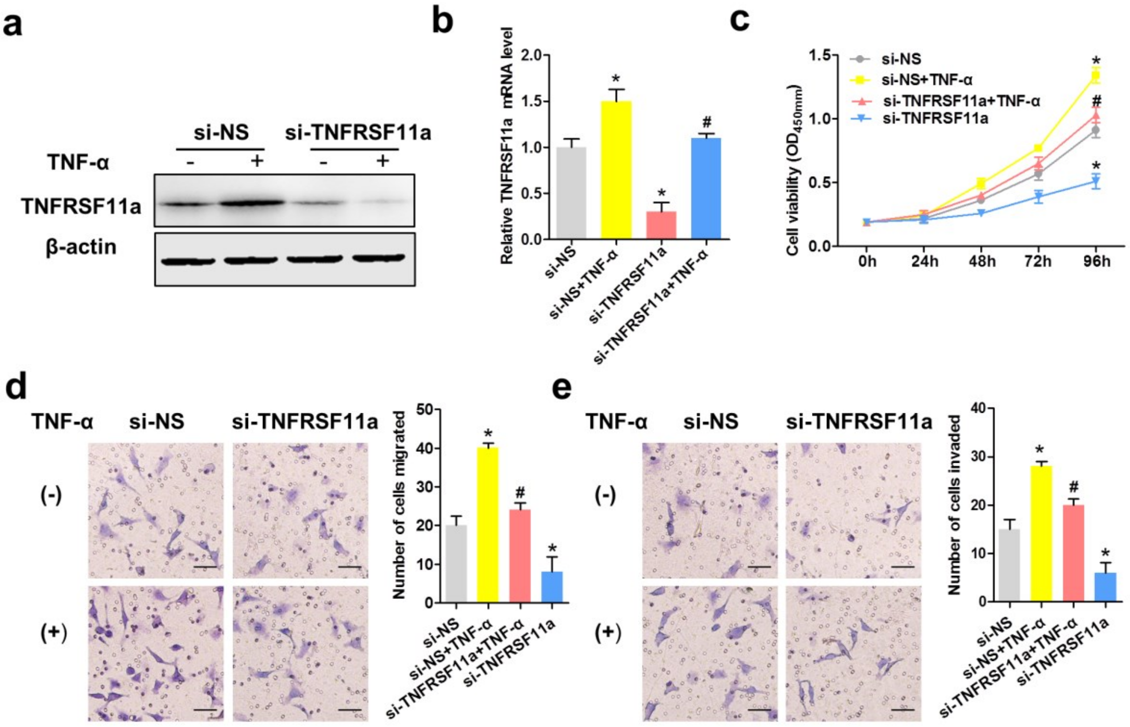

Fig. S2. SiHa cells were transfected with non-specific short interfering RNA (siRNA; si-NS) or tumour necrosis factor receptor superfamily member 11a (TNFRSF11a)-specific siRNA (si-TNFRSF11a) for 24 h and subsequently treated or not with tumour necrosis factor (TNF)-α. TNFRSF11a protein and mRNA expression were analysed by western blotting (A) and quantitative real-time polymerase chain reaction (B). *P<0.05, **P<0.01 vs. the green fluorescent protein adenovirus (Ad-GFP) group. (C) In SiHa cells, stable TNFRSF11a knockdown significantly inhibited cell growth relative to the si-NS treatment during a 96-h period, as measured by the CCK-8 assay. Data represent the means ± standard errors of the means (SEM; n=3). *P<0.05 vs. the si-NS group, #P<0.05 vs. the TNF-α group. TNFRSF11a knockdown altered SiHa cell migration (E) and invasion (F), as shown in Transwell assays. Data represent the means ± SEM (n=3). *P<0.05 vs. the si-NS group, #P<0.05 vs. the TNF-α group.
